# Left ventricular T2 distribution in Duchenne Muscular Dystrophy

**DOI:** 10.1186/1532-429X-11-S1-O51

**Published:** 2009-01-28

**Authors:** Janaka Wansapura, Robert Fleck, Kan N Hor, Wojciech Mazur, Woodrow Benson, William M Gottliebson

**Affiliations:** 1 Cincinnati Children's Hospital, Cincinnati, OH USA; 2 Christ Hospital, Cincinnati, OH USA

**Keywords:** Ejection Fraction, Macro Molecule, Muscular Dystrophy, Duchenne Muscular Dystrophy, Myocardial Fibrosis

## Background

The transverse relaxation time (T2) of water molecules differ between tissues. Because the molecular motion of water is significantly affected by macro molecules, tissue containing fibrous polymers such as collagen has shorter T2 values. Patients with Duchenne Muscular Dystrophy (DMD) develop myocardial fibrosis in the late stage of the disease. The goal of this study was to determine the association of myocardial T2 distribution to the severity of DMD.

## Methods

Twenty six DMD patients and eight normal subjects were studied. T2 maps of the left ventricle were generated using a black blood dual spin echo method. (TE_1_ = 6 ms, TE_2_ = 34 ms) DMD patients were grouped according to the ejection fraction (EF) and the circumferential strain (ε_cc_) as: A: EF ≥ 56%, ε_cc_ ≥ 12%, mean age = 10.5 yrs, B: EF ≥ 56%, ε_cc_ ≤ 12%, mean age = 17.5 yrs, C: EF ≤ 56%, mean age = 17.5 yrs. T2 values were plotted for each subject as a histogram. The normalized mean histograms from each group were compared by the Full Width of Half Maximum (FWHM).

## Results

The FWHM of the T2 histogram was significantly higher in group B and C compared to that of group A and the normal group, indicating significantly high heterogeneity in T2 in Group B and C. FWHM/T2mean is significantly (p < .0001) higher in group B (0.42 ± 0.06) compared to Group A (0.54 ± 0.08). Regression analysis show moderate association between FWHM/T2mean and |Ecc| in group A and B. (Pearson correlation coefficient r = .51).

## Conclusion

The distribution of T2 in the LV of the DMD subjects were remarkably heterogeneous compared to that of normal subjects. These characteristics may suggest early signs of diffused collagen accumulation in the LV in DMD.

**Figure 1:**
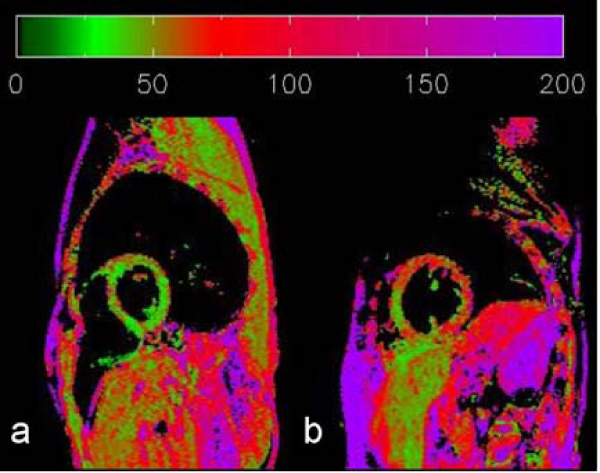
**The T2 maps of a normal (a) and a DMD subject (b) showing the left ventricle in the short axis view**.

**Figure 2:**
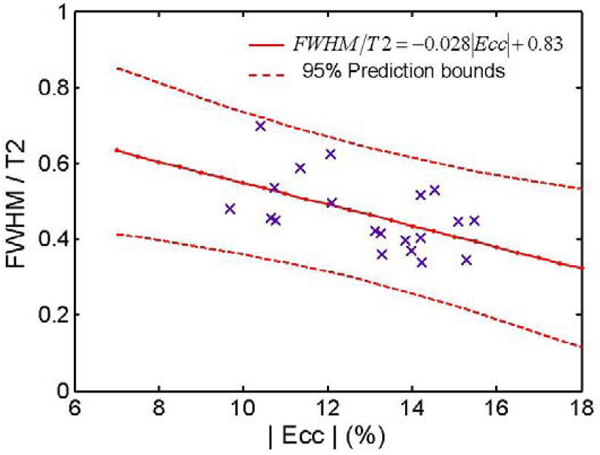
**Association between FWHM/T2mean and ε**_**cc**_ **in group A and B**. Pearson correlation coefficient r = .51.

